# Disability in major depression related to self-rated and objectively-measured cognitive deficits: a preliminary study

**DOI:** 10.1186/1471-244X-7-32

**Published:** 2007-07-17

**Authors:** Sharon L Naismith, Wendy A Longley, Elizabeth M Scott, Ian B Hickie

**Affiliations:** 1 Brain & Mind Research Institute, University of Sydney, Camperdown, Australia; 2 Multiple Sclerosis Society of NSW/VIC, Lidcombe, Australia

## Abstract

**Background:**

Although major depression (MD) is associated with high levels of disability, the relationships between cognitive dysfunction and self-rated disability are poorly understood. This study examined the relationships between self-rated disability in persons with MD and both self-rated and objectively-measured cognitive functioning.

**Methods:**

Twenty-one persons with MD and 21 control participants underwent neuropsychological assessment and z-scores representing deviations from control performance were calculated and averaged across the domains of psychomotor speed, initial learning, memory retention and executive function. Self-ratings of cognitive deficits (SRCDs) were reported on a 6-point scale for overall rating of cognitive change, speed of thinking, concentration, and short-term memory. Disability scores for self-rated physical, mental-health and functional (ie. days out of role) disability were computed from the Brief-Disability Questionnaire and the SF-12 'mental component' subscale.

**Results:**

Persons with MD had a mean age of 53.9 years (SD = 11.0, 76% female) and had moderate to high depression severity (mean HDRS 21.7, sd = 4.4). As expected, depression severity was a strong predictor of physical (r = 0.7, p < 0.01), mental-health (r = 0.7, p < 0.01) and functional (r = 0.8, p < 0.001) disability on the Brief Disability Questionnaire. Additionally, for physical disability, both overall SRCDs and objectively-measured psychomotor speed continued to be independent significant predictors after controlling for depression severity, uniquely accounting for 13% and 16% of variance respectively. For functional disability scores, objectively-measured memory impairment and overall SRCDs were no longer significant predictors after controlling for depression severity.

**Conclusion:**

While depression severity is associated with disability, the contributions of both self-rated and objectively-measured cognitive deficits are substantial and contribute uniquely and differentially to various forms of disability. Efforts directed at reducing cognitive deficits in depression may have the potential to reduce disability.

## Background

As major depression (MD) is the leading cause of life years lived with disability in the developed world [1,2], research designed to investigate the specific determinants of that disability is a high priority. In addition to depression severity, cognitive dysfunction *per se *is likely to be a relevant factor. While cognitive deficits are commonly reported and objectively-measured by neuropsychological testing in MD, the relationship between subjective ratings and objective ratings is unclear. Objectively-measured deficits are variable but are most commonly observed in the areas of processing speed, aspects of executive functioning and, to a lesser degree, in learning and memory [3,4]. A convergence of literature now indicates that these deficits are underpinned by neuroanatomical changes in fronto-subcortical and/or fronto-temporal circuits that suggest a relationship with disease duration, age of onset, regional cerebral blood flow, genetic and clinical risk factors [5,6].

Despite the large number of reports investigating the nature and extent of neuropsychological dysfunction in depression, there is a lack of studies investigating which domains of either self-rated cognitive deficits (SRCDs) or objectively-measured cognitive deficits are associated with disability. While disability is a global construct it can be characterised as including an inability to carry out usual activities or hobbies, a failure to meet daily household or family expectations, having reduced motivation or efficiency at home, school or work, or by having a deterioration in social relationships. It may be necessary to examine these various subtypes of disability as they may be differentially affected by depression and cognitive dysfunction.

Some studies have explored the relationship between depression, disability and cognition in older persons. These have used clinician-ratings or very gross, global measures of cognition that may be insensitive to more subtle forms of cognitive dysfunction. In a large study of community dwelling older Korean residents Kim et al [8] reported that more severe disability levels were associated with physical health, depression and cognitive function as measured by the Mini-Mental State Examination (MMSE)[9]. Additionally, each of these factors were additive in their contribution to disability. In a large sample of Italian geriatric patients after acute hospitalization, Marengoni and colleagues [10] reported that functional disability as determined by impairment in one activity of daily living, was related to depressive symptoms, older age, and poorer cognitive function as measured by MMSE score. In one study of geriatric depression, Kiosses et al [7] reported that disability as measured by instrumental activities of daily living, is related to executive dysfunction and psychomotor retardation. However, executive dysfunction was measured by performance on the initiation/perseveration subscale of a global cognition measure and psychomotor change was clinician-rated. While this is likely suitable for some forms of geriatric depression and dementia, these measures may not be sensitive to the most common executive or processing speed deficits or to the more subtle difficulties in long-term memory retention (ie. measured on neuropsychological testing) typically reported in non-geriatric depressive disorders [3,4].

Emerging literature therefore suggests that depressive symptoms and cognition play some role in determining disability at least in older or elderly persons. However, no known studies have examined these issues using well-standardised neuropsychological instruments which have the capacity to detect even subtle impairments in fronto-subcortical functioning in younger or middle-aged individuals. Further, no known studies have explored the association between a persons' own perception of cognitive deficits, actual neuropsychological performance and the nature and level of disability. In our view, more detailed, sensitive and objective neuropsychological analysis of cognitive impairment, in addition to measurement of the patient's own perception of cognitive impairment in depressed clinical populations is warranted to better elucidate their role in causing disability in this population and to help inform targeted cognitive intervention strategies.

## Methods

### Participants

As reported previously [4], 21 patients meeting DSM-IV criteria for a MD episode were recruited from specialist clinical services in South Eastern Sydney. Twenty-one control participants selected to have similar age, sex and educational backgrounds and screened for history of psychiatric disorder were recruited via newspaper advertisement. Detailed neuropsychological data for control participants have been reported elsewhere [4] and hence, will not be presented in detail here. Additionally, exclusion criteria have been detailed elsewhere [4] and include history of prior head injury, neurological disease, dementia or substance abuse. The study was approved by the Institutional Ethics committee and all subjects gave written informed consent.

### Clinical assessment of severity of depression

Clinical psychiatrists completed DSM-IV criteria and the 17-item Hamilton Depression Rating Scale (HDRS)[11]. To assess the impact of medical burden, the Cumulative Illness Rating Scale [12] was also recorded. This required thirteen major organ systems to be rated on a 5-point scale ranging from '0' (no problem) to '4' (extremely severe problem). Items were summed to give a total score (maximum = 52).

### Disability assessment

Four measures of self-reported disability were employed in this study. All subjects completed the Brief Disability Questionnaire (BDQ), a self-report measure of disability that has been used in psychiatric populations [13]. It assesses disability in everyday activities with responses ranging from '0' (not at all impaired) to '2' (moderately or definitely impaired). Items 1–3 relate to limitations of various forms of 'physical' activity (ie. climbing stairs, bending, carrying groceries), hobbies or daily routine and are summed to yield a score out of 16 to provide a measure of 'physical disability'. Items 4, 5 and 6 were summed and relate more specifically to 'mental-health' related activities such as motivation and efficiency for home, school or work activities and deterioration in social relationships (maximum score = 6) to provide a measure of 'mental-health' disability. Total scores across items 1–6 are considered to indicate 'moderate' and 'severe' disability for score ranges 8–13 and 14–22 respectively. Additionally, participants were asked to estimate how many days over the prior few weeks they were unable to carry out their usual daily activities (maximum = 21) thus providing an estimate of 'functional disability'. Participants also completed the Medical Outcomes Study Short-Form, the SF-12 questionnaire [14] which asks about both disability and wellbeing, thereby potentially providing a more specific measure of mental-health related or 'wellbeing' disability. The mental-disability weighted subscale was computed (mean 50, SD = 10). It has demonstrated test-retest reliability and sensitivity to recovery from depression [14].

### Neuropsychological assessment

In addition to general, global performance on the MMSE [9] (given for descriptive purposes only), participants underwent a neuropsychological test battery to determine objectively-measured neuropsychological deficits. Neuropsychological performance was measured across a number of domains as previously described [4] including:

 Premorbid functioning, assessed by the National Adult Reading Test (NART) [15];

*Psychomotor speed*, as assessed by choice reaction time [16] and Part A of the Trailmaking Test [17];

*Initial learning*, assessed using the Logical Memory subtest of the Wechsler Memory Scale-Revised (WMS-R) Australian version [18](summed stories one and two) and the Rey Auditory Verbal Learning Test [19] (summed trials 1–5);

*Memory retention*, as assessed by percent retention on trial 7, compared to trial 5 of the Rey Auditory Verbal Learning Test and percent retention on delayed versus immediate recall on Logical Memory of the WMS-R; and,

*Executive functioning*, assessed by Part B of the Trailmaking Test [17], the Stroop Color Word Test [20] (items completed in two-minutes, maximum score = 112) and a computer version of the Tower of London test [21].

### Self-rated cognitive deficits (SRCDs)

Persons with MD were asked to rate impairment and/or changes in aspects of their normal/premorbid level of cognitive functioning on a 6-point scale, with '0' being 'no impairment or change' (normal level) to '6' (severe impairment or change for the worse). We evaluated ratings regarding 'overall rating of cognitive functioning' (eg. in 'memory and thinking skills'), 'speed of thinking and responding', 'concentration', and 'short-term memory' (eg. remembering what someone just told you or learning and recalling a set of instructions).

### Statistical analysis and data transformations

For each of the neuropsychological variables, a z-score was calculated for each test based on control group performance [4]. Z-scores, rather than raw scores were used due to the fact that raw scores do not take into account extent of impairment. Z-scores were then averaged across the neuropsychological domains of *psychomotor speed*, *initial learning*, *memory retention *and *executive functioning*.

Since all data were continuous, all univariate analyses used Pearsons product moment correlation coefficients. For examination of the relationships between SRCDs and neuropsychological performance, Bonferroni corrections were employed across the cognitive domains (α = 0.05/4 = 0.0125). Similarly, Bonferroni corrections were employed when examining the relationship between neuropsychological performance and disability (α = 0.05/4 = 0.0125) and SRCDs and disability (α = 0.05/4 = 0.0125). Stepwise multiple regression analyses (F to enter, p < .05) were conducted when both SRCDs and objectively-measured cognitive deficits predicted a form of disability. This was to account for the potentially large amount of shared variance between SRCDs and objectively-measured deficits.

## Results

### Demographic data and disability

As shown in Table 1, the sample comprised twenty-one persons (mean age 53.9, sd = 11.8, 76% female) with moderate-to-severe MD (mean HDRS 21.7, sd = 4.4). On average, patients with MD had 12.9 (sd = 3.7) years of education and an estimated IQ (NART) in the *Average *to *High Average *range. On the BDQ, patients rated *moderate *levels of disability (total mean 12.3, sd = 6.6; physical mean 8.3, sd = 4.9; mental-health mean 4.2, sd = 2.3) and an average of 8.9 days (sd = 7.9) being unable to carry out 'usual activities' in the last few weeks. On the SF-12 'wellbeing' subscale, patients were in the *severe *range of disability (mean 20.9, sd = 12.7).

**Table 1:** Demographic, clinical, and neuropsychological data and z-scores for neuropsychological domains.

	**Mean**	**Standard deviation**
Age, years	53.9	11.8
Sex, % female	76	-
Hamilton Depression Rating Scale	21.7	4.4
Age of onset, years	39.1	16.9
Cumulative Illness Rating	5.2	4.4
*Neuropsychological performance*		
National Adult Reading Test estimated IQ	111.7	7.8
Choice reaction time (secs)	0.77	0.13
Trailmaking, Part A, time (secs)	39.1	15.9
RAVLT^a^, trial 1 to 5 summed score	48.2	11.4
Logical Memory, learning trials	23.5	6.1
RAVLT^a^, percent retention	78.0	29.1
Logical Memory, percent retention	80.8	13.5
Trailmaking, Part B, time (secs)	90.9	27.9
Tower of London, total	10.7	1.9
Stroop, color-word score in 2-mins/112	87.3	21.5
*Z-scores across domains *^#^		
Psychomotor speed	-1.3	1.4
Initial learning	-0.6	0.9
Memory retention	-0.5	1.2
Executive functions	-1.4	1.4

^#^Control data for raw neuropsychological test scores are published in Naismith et al 2006 [4].

^a^RAVLT, Rey Auditory Verbal Learning Test

### Neuropsychological performance

Neuropsychological test data for control participants has been detailed elsewhere [4] and showed that persons with depression had significant performance decrements on tests of choice reaction time, the Trailmaking test (parts A and B), and the Stroop Color-Word Test. While poorer than control participants, performance on a global measure of cognition was good (mean MMSE 28.1, sd = 1.8). In contrast, although variable, z-scores across the more sensitive neuropsychological measures of cognition showed that persons with depression had performance decrements in the mildly reduced range (ie. approximately half a standard deviation lower than controls) on measures of *initial learning *and *memory retention*. They had moderate (ie. greater than one standard deviation) performance decrements on measures of *psychomotor speed *and *executive functioning*. There were no significant relationships between z-scores and age (r = -0.2, ns; r = -0.1, ns; r = 0.03, ns; r = -0.2, ns respectively).

### Self-rated cognitive deficits (SRCDs)

Patients subjectively rated themselves as experiencing moderate levels of overall cognitive dysfunction (mean 3.5, sd = 1.8) and across the specific domains of speed (mean 3.1, sd = 1.9), concentration (mean 3.2, sd = 2.1), and short-term memory (mean 3.3, sd = 2.0). These ratings were not significantly correlated with age (r = -0.1, ns; r = -0.02, ns; r = -0.1, ns; r = -0.1, ns respectively).

### Relationship between SRCDs and objectively-measured neuropsychological deficits

After bonferroni corrections, objectively-measured *memory retention *was moderately related to self-rating of overall cognitive dysfunction (r = -0.6, p < 0.01), as well as concentration difficulties (r = -0.6, p < 0.01), but not to self-rating of speed (r = -0.5, ns) or short term memory (r = -0.5, ns). Objectively-measured *psychomotor speed*, *initial learning*, and *executive functions *were unrelated to any self-ratings of cognition. That is, objectively-measured *psychomotor speed*, *initial learning *and *executive functions *were not associated with self-ratings of overall cognitive functioning (r = -0.4, ns; r = -0.4, ns; r = -0.4, ns respectively), self-rated concentration (r = -0.2, ns; r = -0.5, ns; r = -0.3, ns), self-rating of speed (r = -0.4, ns; r = -0.4, ns; r = -0.5, ns) or self-rating of short-term memory (r = -0.2, ns; r = -0.3, ns; r = -0.3, ns).

#### Predictors of disability

Men tended to report more physical disability (mean 3.8 and 9.5 for males & females respectively), but similar levels of mental and functional disability. However, there were insufficient male participants to draw valid conclusions from this comparison. Table 2 shows that none of the measures of disability were related to age or medical burden. By contrast, most measures of disability were strongly related to severity of depression.

**Table 2:** Correlations amongst neuropsychological domains, self-rated cognitive deficits and disability across physical, mental-health and functional domains.

	**BDQ Physical**	**BDQ Mental-health**	**BDQ Functional**	**SF-12 Wellbeing**
Age	.03	-.17	.08	.37
HDRS	.72**	.70**	.77***	-.49*
Medical burden	.36	.22	.38	.03
*Objectively-measured neuropsychological domains*^#^				
Psychomotor speed	-.63**	-.21	-.16	.08
Initial learning	-.15	.08	-.01	-.04
Memory retention	-.51* ^a^	-.39	-.62**	.16
Executive functions	-.40	.01	.07	.10
*Self-rated cognitive deficits*				
Concentration	.41	.54* ^a^	.47	-.62**
Speed	.54* ^a^	.63**	.60*	-.55**
Short-term memory	.35	.53* ^a^	.60*	-.59**
Overall rating	.73***	.71**	.60*	-.43

HDRS, Hamilton Depression Rating Scale.

BDQ, Brief Disabilty Questionnaire; higher scores indicate more disability.

SF-12 wellbeing, Medical Outcomes Short Form Survey; lower scores indicate more disability.

^#^Negative correlations with BDQ items or positive correlations with SF-12 scores indicate poor neuropsychological performance is associated with greater disability.

*Significant at p < 0.05; **Significant at p < 0.01; ***Significant at p < 0.001.

^a^Non-significant after bonferroni correction for each domain of functioning (0.05/4; α = 0.0125).

### Relationship between disability and neuropsychological deficits

Table 2 shows that after bonferroni correction, physical disability was moderately related to objectively-measured *psychomotor speed*. Functional disability was moderately related to objectively-measured *memory retention*. Neither measure of mental-health disability (ie. BDQ mental-health or SF-12 wellbeing) was associated with any neuropsychological measure.

### Relationship between disability and SRCDs

Table 2 also shows that after bonferroni correction, physical disability was strongly related to patients' ratings of overall cognitive dysfunction. One measure of mental-health related disability (BDQ) was also strongly related to overall SRCD, and also moderately related to a self-rated deficit in speed. The other measure of mental-health related disability (SF-12 wellbeing) was moderately related to self-rated concentration, speed, and short-term memory deficits. Functional disability was moderately related to SRCDs in the areas of speed, short-term memory and overall cognitive functioning.

### Multivariate cognitive predictors of disability

In order to account for the shared variance between overall SRCDs and objectively-measured neuropsychological deficits, multivariate models (stepwise, F-to enter p < 0.05) were constructed after controlling for depression severity using HDRS ratings. Based on results showed in table 2, dependent variables were physical and functional disability (ie. these were the only two disability domains having significant relationships with both SRCDs and objective measures) and after forced entry of HDRS scores, independent predictors were *psychomotor speed *and *memory retention *respectively. Table 3 shows that for physical disability, HDRS was no longer a significant predictor, yet *psychomotor speed *and overall SRCDs independently accounted for 15.7% and 12.5% of the variance in disability scores respectively, with a further 43.9% being shared predictor variance (full model R^2 ^= 72.2%, F = 12.2, df = 3,14, p < 0.001). By contrast, for functional disability depression severity remained the strongest predictor, and after forced entry of this variable (R^2 ^= 40.4%, F = 10.2, df = 1,16 p = 0.006), *memory retention *and SRCDs were no longer significant predictors. This suggests that depression severity and cognitive impairment have differential contributions to physical and functional disability in MD.

**Table 3:** Multiple regression analyses for predictors of BDQ physical and functional disability accounting for depression severity.

	**BDQ Physical**			**BDQ Functional**		
	t	Part R^2a^	p	t	Part R^2a^	p
HDRS, forced entry	0.3	0.2%	ns	3.2	40.4	0.006
Psychomotor speed	-2.8	15.7%	0.014	Not entered		
Memory retention	Not entered			-	-	ns
SRCD, overall rating	2.5	12.5%	0.025	-	-	ns

BDQ physical, F = 12.2, df = 3,14, p < 0.001, R^2 ^= 72.3% (43.9% shared predictor variance), BDQ functional, F = 10.2, df = 1,16, p = 0.006, R^2 ^= 40.4%

HDRS = Hamilton Depression Rating Scale forced entry in the regression model 1

^a ^Part R^2 ^= part correlation squared representing unique predictor variance

ns = non-significant (F to enter p < 0.05) predictor in stepwise multiple regression analyses

## Discussion

This study explores the relationships between various measures of self-rated disability, objectively-measured cognitive impairment, and self-rated cognitive impairment in a clinical population of persons with moderate to severe depression. As expected, depression severity is a strong predictor of disability. However, it is also clear that physical disability is *equally *strongly predicted by self-rated overall cognitive dysfunction, and also moderately predicted by objectively-measured psychomotor slowing. These findings are consistent with the study by Kiosses et al [7] where psychomotor retardation was a predictor of instrumental activities of daily living in geriatric depression. They also add to these findings suggesting that depression severity, perception of cognitive deficits and psychomotor slowing contribute uniquely to this form of disability.

This study is novel in this area as we also apportioned disability into 'mental' and 'functional' components. In doing so we found that 'mental-health' disability or 'wellbeing' is also strongly predicted by depression severity, but equally strongly predicted by self-rated overall cognitive functioning, and moderately predicted by self-ratings of concentration, speed, and short-term memory changes, but not any measures of objectively-measured cognitive impairment. An important alternative to the traditional division of 'mental' versus 'physical' forms of disability, is the use of the global notion of 'functional' disability. This was defined by the amount of time (over the prior few weeks) that patients were unable to carry out their usual activities. Again, while this measure of functional disability is predicted most strongly by depression severity, it is also related to self-rated overall cognitive functioning, self-rated speed and short-term memory impairments, and to objectively-measured memory retention impairment. However, for functional disability the association between cognitive dysfunction and disability may be mediated by depression severity. Overall, while this small clinical sample offers only limited opportunity for multivariate analyses, preliminary results suggest that both objective and subjective measurement of cognitive dysfunction differentially relate to various forms of disability: physical disability appears to relate to cognitive dysfunction regardless of depression severity whereas functional disability is largely related to depression severity. Further studies would be warranted to explore these findings more systematically in larger samples.

A secondary aim of this investigation was to examine the concordance between results of formal neuropsychological testing and the degree of SRCDs. To our knowledge, these associations have not been examined previously in persons with depression. Our findings suggest that only memory retention is well predicted by some measures of SRCDs, while speed, learning and executive dysfunctions are not well predicted by perceived changes. This is an interesting finding since it raises questions regarding what self-ratings of cognitive impairment actually represent. This may suggest that SRCDs capture a different component of disability or that in real-life, less ideal testing situations where the environmental demands on cognition are more challenging, cognitive impairment may manifest quite differently to that recorded by neuropsychological assessment. Further exploration of these issues may be enhanced by larger studies using a scale of self-reported deficits validated for use in persons with depression.

In terms of the more sensitive and specific neuropsychological assessment findings, this study has shown direct links between measures of both 'physical' and 'functional' disability and objectively-measured psychomotor slowing and impaired memory retention in patients with MD. In our previous studies we have linked these two aspects of cognition and the size of the caudate nucleus and hippocampus respectively [5,6]. Our current study extends our previous work by suggesting that the inter-relationships between neurobiological underpinnings and neuropsychologically-verified cognitive deficits also differentially and uniquely relate to disability in MD.

Importantly, interventions focused on remediation of these underlying cognitive deficits, may also help to prevent disease-specific brain changes and consequent disability. Implementation of psychosocial, cognitive and/or vocational programs that particularly target difficulties with psychomotor speed, memory or 'perception' of cognitive deficits may not only help to improve cognition and relieve symptoms but also to reduce the disability associated with MD. Such strategies may involve the use of adaptive or compensation strategies (eg. use of diaries, using strategies to enhance learning and recall) or formal cognitive remediation or neurorehabilitation programs targeting fronto-subcortical brain functioning.

## Conclusion

Overall, these findings suggest that the disability experienced by persons with MD is strongly linked to both depression severity and to self-reported cognitive deficits. In particular, an 'overall' self-report of cognitive deficits predicts most forms of self-rated disability as well as depression severity. Additionally, both physical disability and functional disability, as defined by inability to carry out tasks or by days out of role, were related to objectively-measured psychomotor slowing and impaired memory retention. These findings suggest that while disability is largely related to illness severity, cognitive dysfunction also plays a critical role and should be targeted for cognitive intervention or neuropsychological rehabilitation. These findings may therefore be particularly relevant to workplace and vocational programs, and to research protocols that are focused on global health and functional outcomes. Lastly, given that a simple and quick self-rated measure of overall cognitive functioning has been shown to predict disability as well as a lengthy clinical assessment of depression in this sample, self-rated cognitive impairment in MD deserves further exploration as a screening method.

### Limitations and future research

In this study, the scale used for assessing perceived cognitive deficits is not a widely-used instrument and the psychometric properties have not been established. Additionally, disability ratings and perceived cognitive changes were assessed via self-report. Both ratings may, therefore, be affected by common patterns of negative responding in persons with depression. However, most measures of neuropsychological performance were poorly related to self-reported cognitive functioning and yet objectively-measured speed and short term memory still predicted certain key aspects of disability suggesting that brain changes play some role in disability. These issues can be explored in studies using objective ratings of disability and/or informant reports. Due to the small clinical nature of this study, future studies may also be enhanced by a larger sample size and by more extensive exploration of multivariate relationships. This may also allow researchers to more thoroughly examine the differential contributions of depression severity and cognitive change.

## Abbreviations

MD = major depression

MMSE = Mini-Mental State Examination

SRCDs = self-reported cognitive deficits

## Competing interests

The author(s) declare that they have no competing interests.

## Authors' contributions

SN, ES and IH were involved in study conception and design and personally conducted the study and provided clinical (ES, IH) and neuropsychological assessments (SN). SN was responsible for data analysis and manuscript preparation. WL designed the self-report rating scale used in this study and provided extensive intellectual input into data analysis and interpretation. SN, WL and IH were critically involved in manuscript drafting and revision. All authors have given final approval for publication.

## Pre-publication history

The pre-publication history for this paper can be accessed here:


